# Conducting Digital Health Care Research: Document Analysis of Challenges Experienced During Intervention Development and Feasibility Study Setup of an Internet-Administered Intervention for Parents of Children Treated for Cancer

**DOI:** 10.2196/26266

**Published:** 2021-10-08

**Authors:** Joanne Woodford, Mathilda Karlsson, Josefin Hagström, Ylva Hägg Sylvén, Kajsa Norbäck, Helena Grönqvist, Louise von Essen

**Affiliations:** 1 Healthcare Sciences and e-Health Department of Women’s and Children’s Health Uppsala University Uppsala Sweden

**Keywords:** parents, internet-administered cognitive behavior therapy, low intensity CBT, feasibility study, challenges, digital healthcare research

## Abstract

**Background:**

The design and conduct of research to develop, test, and evaluate complex health care interventions is challenging. Although the existing literature describes key challenges associated with the design and conduct of definitive (evaluation) trials, there is a lack of information concerning specific challenges associated with the intervention development phase and setup of feasibility studies. In particular, the literature is scarce concerning the challenges associated with conducting digital health care research, such as research on internet-administered interventions and research using digital features to support the execution of study procedures (eg, recruitment, consent, retention, and data collection and management). This study is conducted in the context of the intervention development and feasibility study setup phases of an internet-administered, guided, low-intensity cognitive behavioral therapy–based intervention for parents of children previously treated for cancer.

**Objective:**

The aim of this study is to explore the challenges experienced during the development phase of the internet-administered intervention and digital features to support the execution of the study procedures and a feasibility study setup.

**Methods:**

To explore the key challenges experienced, we conducted a document analysis of written records from all study meetings held by the research team (meeting minutes) between June 7, 2018, and January 10, 2020, guided by a thematic analysis approach. Furthermore, discussion groups with members of the research team were held to develop a more detailed understanding of the key challenges experienced. Methods and results are reported in accordance with the relevant items from the Standards for Reporting Qualitative Research checklist.

**Results:**

Six main themes were identified: decision-making and communication, expertise, external constraints, flexibility, planning and scheduling, and technical constraints.

**Conclusions:**

Significant challenges were experienced during the intervention development and setup phases of the feasibility study. Implications are discussed to inform future design, conduct, and planning of internet-administered intervention development and feasibility studies, especially within the context of digital health care research.

## Introduction

### Background

Clinical trials are essential to inform evidence-based health care [1]. However, clinical trials are costly and resource-intensive for both researchers and funders [2], and approximately 85% of research investment is wasted [3]. Examples of research waste include asking the wrong research questions [4], using inappropriate study designs and methods [5,6], poor and biased reporting [7], and underreporting [8]. There are a number of inefficiencies related to clinical trial conduct that can lead to research waste, especially given that the design and efficient conduct of clinical trials is challenging [9] and operationally complex [10,11]. Challenges include the creation and management of trial procedures and materials; communication with multiple stakeholders; ethical and regulatory requirements; the recruitment, training, and turnover of trial personnel; budget management; the recruitment and retention of trial participants; and data monitoring and assurance of data quality [10-14].

While numerous barriers have been identified in the successful conduct of clinical trials [12], there has been less focus on sharing experiences and lessons learned by trial teams [10]. Publications tend to focus on trial outcomes, with little reporting on trial conduct [11]. Consequently, there is scarce evidence to inform decisions concerning the management of clinical trials [1]. Furthermore, in contrast to the literature focused on the conduct of definitive (evaluation) trials, there is a lack of literature concerning the challenges of conducting intervention development research and pilot and feasibility studies, following the Medical Research Council (MRC) framework for the development and evaluation of complex interventions [15]. Indeed, there is limited guidance on how to design and conduct feasibility studies, leading to poor study design and reporting [16,17]. Given that many preparatory development [18,19] and clinical, methodological, and procedural uncertainties require testing [20] before progressing to a definitive trial, development and feasibility phases are substantial works in and of themselves, with findings often underreported [16,17].

In the context of this feasibility study (ENGAGE study: ISRCTN 57233429; ISRCTN 18404129) [21], the internet-administered low-intensity cognitive behavioral therapy (LICBT) intervention (the EJDeR intervention) is delivered on the U-CARE-portal, hereafter referred to as the Portal [22]. The Portal is designed to deliver internet-administered cognitive behavioral therapy interventions and support the execution of study procedures, for example, randomization, web-based informed consent, and data collection [23]. Digital technologies (eg, technologies using the internet) are facilitating the delivery of health care worldwide [24,25]. Indeed, internet-administered interventions have been posited as a solution to the global mental health crisis and to help overcome significant barriers to treatment access (eg, geographical and resource barriers) [26]. The promise of digital technologies to deliver mental health care interventions has been further amplified by the current COVID-19 pandemic [27], given the negative psychosocial consequences of the pandemic itself [27,28] and the ability to facilitate access to mental health care [29]. However, while the evidence base for internet-administered psychological interventions is well-established [30], many publicly available interventions delivered via digital technologies are not evidence-based [31].

Furthermore, digital technologies are being increasingly incorporated into the design and execution of health care research, for example, to facilitate recruitment, enhance retention, and collect data [24,25,32,33]. The use of digital technologies in health care research has intensified during the COVID-19 pandemic in an attempt to continue health care research in the absence of in-person contact [34]. Digital health care research is associated with reduced trial costs, improved trial efficiency [35,36], and recruitment of more diverse populations [25,34]. As such, the use of digital features to execute health care research is likely to continue to grow beyond the pandemic [34]. However, at present, challenges related to the conduct of digital health care research are less well documented [25,32] and focus on topics such as patient privacy and confidentiality, adequate infrastructure, data accuracy and integrity, and user acceptability [25,37,38]. Some challenges are beginning to be addressed by the development of new ethical and regulatory standards and increased provision of guidance for investigators in the use of digital technologies [25,37,38]. However, the literature remains in its infancy, and there is a need for researchers to publish their experiences in conducting digital health care research [25].

### Context: The ENGAGE Feasibility Study

Globally, approximately 300,000 children are diagnosed with cancer each year [39], and cancer remains a leading cause of death in children worldwide [40]. Typically, parents are the primary source of support for children with cancer and report significant negative psychological [41-43] and socioeconomic impacts [44-47]. Mental health difficulties are reported after cancer treatment [42,43,48] and years after the end of treatment [42,49]. However, parents of children treated for cancer report an unmet need for psychological support [50-52]. To improve access to evidence-based psychological support, innovative solutions are being developed worldwide [53]. One such solution is the provision of guided internet-administered LICBT, which may help improve access to psychological support for parents of children treated for cancer.

Given the promise of internet-administered LICBT, we have adopted the MRC complex interventions framework [15] to develop an internet-administered LICBT intervention (the EJDeR intervention) tailored to the specific needs of parents of children previously treated for cancer [54]. Significant previous research [41,42,49,55-57] has informed the development of the EJDeR intervention alongside multiple stakeholders, including parent research partners (PRPs), clinical psychologists, software developers, and pediatric oncologists. The EJDeR intervention is delivered on the Portal and includes written, film, audio content, videoconferencing, and in-portal email guidance from an e-therapist [54].

The objectives of this study are to explore the challenges experienced during (1) the development phase of the internet-administered intervention and digital features to support the execution of the study procedures and (2) a feasibility study setup [21,58]. To explore the key challenges experienced, we conducted a document analysis of written records from all study meetings held by the research team (meeting minutes) between June 7, 2018, and January 10, 2020, guided by a thematic analysis approach. Furthermore, discussion groups with members of the research team were held to develop a more detailed understanding of the key challenges experienced.

## Methods

The methods and findings are reported in accordance with relevant items from the Standards for Reporting Qualitative Research checklist [59].

### Qualitative Approach and Research Paradigm

A document analysis [60] of study meeting minutes was guided by a thematic analysis approach [61]. Document analysis was considered suitable given that it allows an examination of contextual and background information to provide historical insights into the challenges experienced by the ENGAGE research team and provides a way of tracking challenges over time [60].

### Researcher Characteristics and Reflexivity

Document analysis was primarily conducted by 2 members of the research team (MK and JW). MK is a female research assistant with a Bachelor of Sport Science who joined the research team toward the end of the study setup phase and was therefore able to conduct the analysis from an *outsider* perspective. MK was trained in using thematic analysis by JW. JW is a female researcher with a PhD in psychology with experience in conducting qualitative research. JW is a coinvestigator, supervisor of the study coordinator, and research assistant in the research team and has been a member of the research team from the beginning of the study setup phase. All manuscript authors are, or have been, members of the research team.

### Context

All meeting minutes were taken by the ENGAGE study coordinator, or a substitute, and meetings were held at the Department of Women and Children’s Health, Uppsala University, Uppsala, Sweden. In most cases, all core members of the research team were in attendance, including the principal investigator (LVE), researchers (JW and HG), study coordinator (KN), Portal development team coordinator (YHS), and research assistants (JH and MK). Occasionally, wider research team members, such as software developers, licensed psychologists, and student interns, attended meetings. Meetings were held weekly and scheduled for 2 hours. Meeting minutes were circulated to research team members for approval after the meeting and saved on a shared folder and thus visible to all research team members.

### Ethical Issues

Ethical approval was not deemed necessary as the study analyzed documentary data only.

### Data Collection

All meeting minutes (N=78) from ENGAGE study meetings conducted between June 7, 2018, and January 10, 2020, were included to account for meetings concerning the development of the intervention, the development of digital features to support study execution, and the concurrent setup of the feasibility study. The number of meeting minute pages ranged from 1 to 6 and the word count range was 85-2350. Meeting minutes were designed to document (1) progress toward specific study milestones, (2) problems or challenges arising, (3) decisions, and (4) actions moving forward.

### Data Analysis

**Figure 1:**
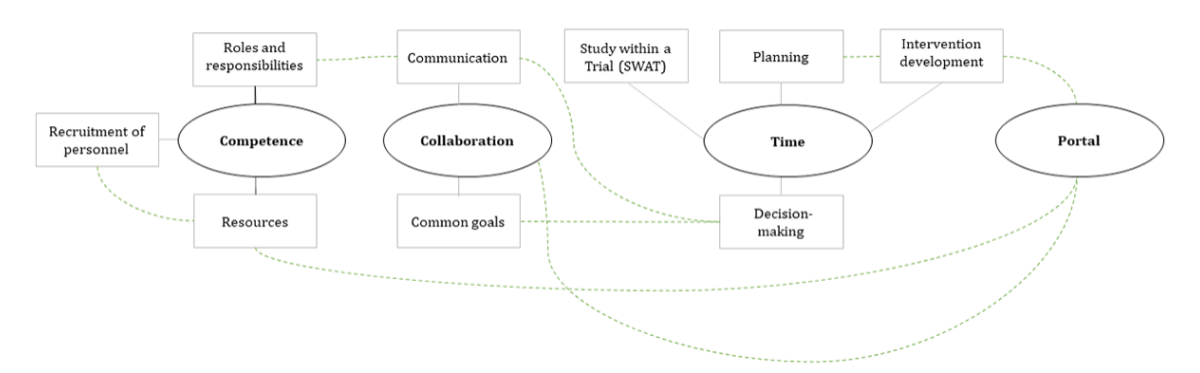
Initial thematic map. Ovals represent main themes and rectangles represent subthemes within main themes. Solid gray lines illustrate connections between main themes and subthemes. Dotted green lines illustrate where main themes and subthemes overlap with other main themes and subthemes.

Given that documents cannot be considered to be completely accurate recordings of past events [60], throughout the analysis process, MK and JW actively reflected upon the meaning of the meeting minute content and how this was related to challenges experienced by the research team. Guidelines for conducting a reflexive thematic analysis followed [61]. An inductive approach was adopted with codes and themes driven by the data [62]. The analysis of meeting minutes took place between January and May 2020. Meeting minutes were read multiple times by MK to enable familiarization with the data set as a whole. MK identified initial codes across the data set to begin organizing the data. To enhance rigor, initial codes were discussed in weekly meetings with JW. After initial coding across the data set was complete, MK sorted initial codes into initial main themes and subthemes within main themes, with continued weekly discussions with JW to establish initial consensus and refine the main themes and subthemes [61]. Next, the initial thematic map (Figure 1) was presented to the research team (HG, JH, JW, KN, LVE, MK, and YHS) for feedback in a face-to-face discussion group held on February 14, 2020 (120 minutes). During the discussion group, the initial thematic map was presented, and discussions were held concerning whether the initial main themes and subthemes reflected the main challenges experienced. One major discussion concerned whether the Portal was a main theme or an element of all the main themes.

**Figure 2:**
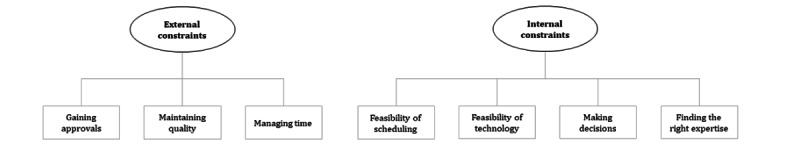
Refined thematic map. Ovals represent main themes and rectangles represent subthemes within each main theme. Solid lines illustrate connections between main themes and subthemes.

Subsequently, a refined thematic map (Figure 2) was developed by MK with continued weekly discussions with JW and applied across the data set to ensure a good fit to the data. A second face-to-face discussion group (120 minutes) was held on March 5, 2020, to present the refined thematic map to the research team (HG, JH, JW, KN, LVE, MK, and YHS) for further discussion and feedback.

**Figure 3:**
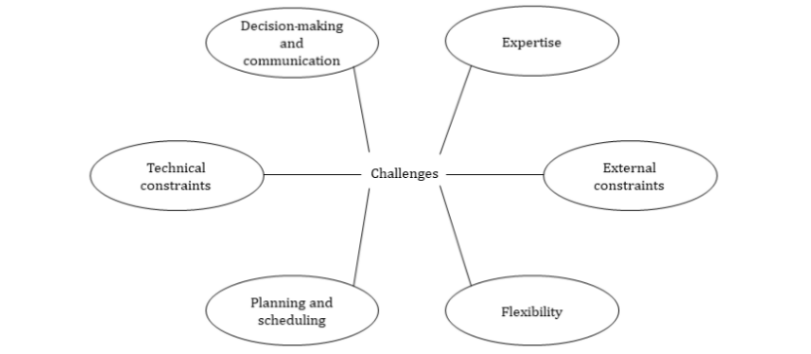
Final thematic map.

On the basis of feedback received in the second discussion group, the main themes and subthemes were further revised by MK and JW, and a final thematic map was developed with six main themes and no subthemes (Figure 3). The thematic map, alongside descriptions of each main theme, was sent to the wider research team via email for final feedback and approval, including the provision of salient examples of challenges missing from descriptors.

### Trustworthiness

To enhance the trustworthiness of the analysis, a number of strategies were adopted as follows: (1) member-checking with research team members to test that the main themes and detailed descriptors were recognized [63], (2) use of thematic maps to explore the main theme and subtheme connections during the analysis process, (3) repeated returning to the whole data set to check the adequacy of each thematic map, and (4) member-checking and (5) peer debriefing with initial codes and subsequent thematic maps reviewed and discussed by MK and JW throughout the analysis process.

## Results

### Overview

The thematic analysis resulted in a three-step process, an initial thematic map (Figure 1), a refined thematic map (Figure 2), and a final thematic map (Figure 3). Textbox 1 presents the content of the final main themes.

**Final themes and descriptors of challenges.**

**Descriptors of challenges identified in main themes**

- Decision-making and communication
  Involving all members of the research team in making certain decisions. Considering the different preferences of research team members
  Communicating between the research team and the portal development team concerning technical requirements, time estimation, and planning
- Expertise
  Identifying and recruiting study personnel with the necessary expertise and experience to design and set up the feasibility study
  External constraints
  Uncertainties regarding time taken for tasks to be completed by contracted personnel outside of the research team and time taken for public authorities to make decisions and provide approvals
- Flexibility
  Changing intervention requirements, elements of the study protocol, and associated study documentation
- Planning and scheduling
  Identifying required tasks to complete
  Estimating time to complete tasks
  Deciding the order in which tasks should be completed, especially concerning task interdependency
- Technical constraints
  Managing within the constraints of available technical resources and known limitations of the Portal
  Identifying necessary technical requirements on the Portal

### Decision-making and Communication

Decision-making was a significant challenge during the development of the intervention and digital features to support study execution, and the feasibility study setup phase, resulting in time inefficiencies and a delay to study start. A particular challenge was related to occasions in which the entire research team was involved in the decision-making process. Group decision-making resulted in the time taken for the principal investigator or a researcher to make a final decision being longer than was always necessary. A tension was identified concerning the desire to build and maintain a supportive team environment versus the need for research team members with more experience and leadership responsibilities to make quick decisions. This resulted in examples from the meeting minutes of the group decision-making process taking weeks, or sometimes months, before a final decision was made by the principal investigator. Group decision-making was particularly challenging when research team members had different preferences, opinions, and different levels of experience and expertise, resulting in difficulties in reaching a consensus. One example was deciding on a name for the intervention, whereby several opinion polls were made over a number of weeks before a final decision was made.

Communication was also identified as a challenge that contributed to difficulties in the decision-making process. Challenges regarding communication were particularly exacerbated in situations where research team members had different areas of expertise. A prevalent example concerned communication between researchers and the Portal development team. For example, during the development of the intervention and digital features to support study execution, the research team had many technical requirements. However, sometimes, there was a mismatch between researchers and the Portal development team in their understanding of technical requirements (eg, how interactive homework exercises were presented on the Portal). Furthermore, difficulties were identified concerning time estimation and subsequent planning. For example, the Portal team at times underestimated the length of time to deliver a technical requirement, or technical requirements were changed by the research team, subsequently impacting the study time plan. In addition, researchers sometimes held unrealistic expectations concerning delivery time for a new requirement, negatively impacting the study time plan. Difficulties with communication were more prevalent in the study setup phase. During the study setup phase, a software developer from the Portal team began to attend research team meetings, helping to avoid misunderstanding and clarifying requirements.

### Expertise

Identifying personnel with the required research and clinical expertise was challenging. One challenge related to the recruitment of research team personnel with the correct competencies and experience, for example, prior experience of trial management, design and conduct of feasibility studies, and/or experience of internet-administered intervention development. For example, multiple recruitment rounds were held to recruit a postdoctoral researcher to work full-time in the study; however, no candidates with the correct expertise were found. In addition, retention of research team personnel (eg, research assistants) was raised as a difficulty, for example, staff turnover was experienced due to short-term temporary contracts and the uncertainty of future employment being tied to study funding. As such, difficulties were experienced needing to replace temporary personnel and provide training to new research team members, taking significant time and further impacting the study time plan. This was especially problematic as it was difficult to recruit research assistants with prior experience of working in similar research environments, and thus the need for training was understandably greater.

Another challenge related to expertise concerned the further development and refinement of the intervention material. Difficulties were experienced in identifying a licensed psychologist with specific experience in writing LICBT interventions in Swedish to finalize the intervention material. Ultimately, 2 English-speaking experts in LICBT were engaged in later iterations of the intervention material after feedback from PRPs. However, this resulted in the need for translation into Swedish, back-translation into English, and cultural adaptation of the intervention material, which further delayed intervention development.

Challenges were also experienced when expectations for the required expertise changed. One example is related to illustrations for the EJDeR intervention, where illustrations were initially developed by a research team member with some experience in using design software. However, it was later decided to engage an external company with specialist experience of developing illustrations for websites and mobile devices. This decision was made to enhance the quality of the EJDeR intervention alongside considerations concerning resource allocation, as illustration design is time consuming and the skillset of the member of the research team was better used elsewhere.

### External Constraints

During the development of the intervention and digital features to support study execution and feasibility study setup phases, the research team liaised with public authorities (eg, the Swedish Ethical Review Authority, the Childhood Cancer Registry, and the Swedish Tax Agency), external companies (eg, professional illustrators), and external contractors (eg, licensed psychologists and e-therapists). One challenge relates to the time taken by public authorities to make decisions and provide approval. A specific example related to the submission of an ethical amendment to the Swedish Ethical Review Authority regarding the implementation of a study within a trial [58] embedded in the ENGAGE feasibility study. The study within a trial was approved; however, the authority raised concerns regarding previously approved parts of the study design (eg, an opt-out recruitment procedure involving telephone reminders to parents who do not actively decline study participation). Raising concerns regarding a previously approved application was potentially because the Swedish government replaced regional ethical review boards with a single national review authority during the study setup phase, meaning applications and requests for amendment were no longer necessarily reviewed by the same local authority. This resulted in additional delays, with the research team needing to respond to the authority to further justify the use of an opt-out procedure. Additional delays were experienced by external companies and contractors, for example, not working at full capacity during the summer of 2019 (eg, taking a summer vacation). Another delay related to external factors was experienced when Uppsala University upgraded their servers, resulting in members of the Portal team being allocated to mitigate risks to the Portal during the upgrade and thus unable to prioritize technical requirements for the study and intervention.

### Flexibility

A further challenge was related to the development of the intervention and digital features to support study execution and the feasibility study setup taking place concurrently, and the subsequent need for flexibility. For example, during the intervention development phase, multiple changes and continued improvements were made to the intervention content, language, and overall design (eg, font, color palette, logo design, professional illustrations, and layout). However, any change to language and design had a subsequent impact on other study components, for example, participant information sheets. It was perceived that there were many interrelated *moving parts* and any detail changed in the intervention or feasibility study resulted in a *snow-ball effect* on multiple other intervention and study components. A further example relates to study documentation. While refining the intervention, specific study terminology was still under development, for example, intervention name, e-therapists being referred to in the study as *parent guides*, the structure of the intervention, module, and chapter titles. During the feasibility study setup phase, study documentation was under development (eg, case report forms, data management, SMS text message and email reminder protocols and reminder content, standard operating procedures, and the study handbook). However, each terminology change resulted in revisions to all study documentation. Another example related to the study information video, which had to be rerecorded after numerous changes were made to the study information sheets based on changes to terminology used in the intervention. Research team members raised that these challenges seemed related to setting up a feasibility study when the intervention had not yet been finalized. The research team needed to be flexible and adaptable during this process and keep track of any detail and subsequent impact on other elements of the intervention and feasibility study.

### Planning and Scheduling

Another challenge is related to the overall timeframe and associated planning and scheduling of tasks. For example, given the aforementioned challenges with delays in intervention development, meeting minutes documented difficulties scheduling specific events, such as training e-therapists, or presenting the intervention to PRPs for feedback. On several occasions, e-therapist training was rescheduled due to delays in intervention development; for example, finalizing the written intervention material and some technical features, such as videoconferencing, taking longer than initially anticipated to develop and test. Furthermore, time for task completion was often underestimated, which was discussed at times as being related to expertise. This could be observed by how several research team personnel had no prior experience of working within a similar research environment, resulting in some tasks taking longer than anticipated. Planning and scheduling were also described as generally difficult given the interrelatedness of intervention development and feasibility study setup tasks. For example, any changes made to the intervention affected the entire *planning chain.*

A further complexity regarding planning and scheduling related to the different project management tools used by the research team versus the Portal team. For example, the research team used a Gantt chart, mainly focused on research-associated tasks, alongside Microsoft To Do, to enable task breakdown and allocation of tasks between research team members. However, the Portal team used Atlassian Jira Software Server Version 8.5.1, a software development tool used by agile teams to plan, track, and release software features. At times, this was experienced as a challenge as while the research team tracked overall technical feature development in the Gantt chart, this was at times out of sync with more detailed software development planning in Jira Software Server. During the study setup phase, the developer team began to report updates to the research assistant responsible for the Gantt chart each week, which improved planning. However, research team members discussed how a more integrated solution may have facilitated scheduling and planning, especially because of the detail and complexity involved in the development of new digital features for both the intervention and support study execution.

### Technical Constraints

A challenge frequently mentioned in meeting minutes pertained to the availability of technical resources, such as the Portal team’s time and the existing functionality of the Portal. The existing Portal did not have all the digital features required to deliver the internet-administered intervention or to support the execution of the feasibility study. Meeting minutes documented numerous new or adjusted intervention features, for example, (1) color palette change, (2) e-therapist notifications via email and SMS text message when participants send internal messages or submit chapters or homework exercises, (3) font selection, (4) intervention display to allow e-therapists to tailor the intervention, (5) carrousel feature to enhance library navigation, (6) printable PDF documents of homework exercise, (7) tab-based intervention view, and (8) videoconferencing. Meeting minutes also document new digital features required to support study execution, for example, (1) character limitation removed for SMS text messages sent via the Portal; (2) customized study registration process, including the use of recruitment ID to identify the source of recruitment; (3) individual preferences for reminders (eg, email, SMS text message, post, or telephone); (4) new home page to facilitate study sign-up and log-in for existing participants; (5) newsletter scheduling based on parents’ progress in the study; (6) opt-out procedure for participants declining study participation; (7) personalization of reminders (eg, use of first name); (8) reporting features on intervention use, newsletters, reminders, and suicide alerts; and (9) study-specific technical help-texts throughout the Portal.

Some new digital feature requirements were known from study conception (eg, included in the grant application and study protocol), for example, the newsletter, individualized reminders, and opt-out procedure. However, other digital requirements, especially in relation to the intervention, were not planned and arose during the intervention development phase. During the discussion groups, it was raised that as it was very difficult to know all Portal requirements in advance, there was a need for good communication between the research team and Portal team. Some research team members discussed how their lack of prior knowledge of the Portal, especially in relation to how interventions were delivered, resulted in underestimations or differing expectations concerning the amount of technical and aesthetic changes required. Furthermore, the research team did not always have a clear understanding of how much work might be involved in developing a new technical feature or changing an existing feature. Meeting minutes also listed several occasions when new digital features, or requested changes to existing digital features, took longer than anticipated to develop and test. One reason for the difficulties in time estimations is related to the existing software architecture of the Portal. As such, the development of new digital features, or making changes to existing digital features, could have unintended or unanticipated consequences on other existing features on the Portal, and subsequently impact other studies running on the Portal.

## Discussion

### Principal Findings

This paper describes the challenges experienced during the development phase of the internet-administered intervention and digital features to support the execution of the study procedures and setup of the ENGAGE feasibility study. To summarize the main findings, a document analysis of meeting minutes adopting a thematic analysis approach and subsequent research team discussions resulted in six main themes as follows: (1) decision-making and communication, (2) expertise, (3) external constraints, (4) flexibility, (5) planning and scheduling, and (6) technical constraints.

### Comparison With Prior Work

To provide an overview of prior work exploring challenges experienced in traditional and digital health care research and software development projects, we present each of the main themes and descriptors of challenges identified alongside similar research findings from others (Table 1). We included 10 publications [64-73] in Table 1, which have not yet been mentioned.

**Table 1:** Final main themes, descriptors of challenges identified, and a summary of similar research findings.

Main themes	Descriptors of challenges identified in main themes	Similar research findings from others
Decision-making and communication	Involving all members of the research team in making certain decisions Considering the different preferences of research team members Communicating between the research team and Portal development team concerning technical requirements, time estimation, and planning	Challenges in communicating between the principal investigator and the research team [9] Cultural interference in group decision-making [64,65] Insufficient time from the principal investigator [9] Lack of arena for solving conflict [66] Misunderstanding project goals [67] Difficulties with communication between software personnel and nontechnical personnel (eg, different perceptions, knowledge, and experience) [66,68]
Expertise	Identifying and recruiting study personnel with the necessary expertise and experience to design and set up the feasibility study	Limited access to project personnel with the relevant expertise and knowledge [9,25,37,68] Poor retention of personnel [69] Time needed to train personnel [9,25]
External constraints	Uncertainties regarding time taken for tasks to be completed by contracted personnel outside of the research team and time taken for public authorities to make decisions and provide approvals	Time taken for ethical and regulatory approval from public authorities [69,70] Lack of adequate regulatory and legal guidance [23,25,37,38]
Flexibility	Changing intervention requirements, elements of the study protocol, and associated study documentation	Adaptations needed to allow digital tools to be used in research [25,36] Complexities of study documentation development [9] Lack of understanding of requirement complexity [68]
Planning and scheduling	Identifying required tasks to complete Estimating time to complete tasks Deciding the order in which tasks should be completed, especially concerning task interdependency	Conflicting priorities [68] Complexities of forecasting and planning project budgets [71] Poor or unrealistic project planning [9,68] Need to involve multiple stakeholders in the planning [25,37,38] Time taken to develop high-quality documents and data collection tools [68] Use of collaboration tools [66]
Technical constraints	Managing within the constraints of available technical resources and known limitations of the Portal Identifying necessary technical requirements on the Portal	Shared an understanding of software requirements [23,66,68] Data privacy and security [23-25,37,38,72,73] Access to adequate infrastructure [23,25,37,38]

One interesting challenge was identified in relation to the group decision-making process affecting the study timeframe. Research concerning cultural expectations and decision-making indicates that, within the Swedish culture, there are high expectations for shared authority and decision-making between managers and personnel [64]. Generally, the workplace is less hierarchical with decisions and responsibilities shared and decisions made in larger groups [65]. However, research suggests that more unilateral or directive decision-making may be required when there are significant time pressures and critical deadlines and with more novice team members [74]. While adopting group decision-making processes may help to maintain good relationships and is more in line with cultural expectations, this was at tension with the need for more unilateral and directive decision-making, given the time critical nature of the research. Indeed, one reason for failing clinical trials may pertain to a lack of structured *business-like* trial management [12].

A related challenge concerned the difficulties in recruiting experienced study personnel. Difficulties with recruitment and inadequate training of study personnel have been identified in the literature as a key inefficiency in successful trial delivery [9]. Despite multiple recruitment attempts, it was not possible to recruit a postdoctoral researcher with the required competencies to work on the study. However, competent trial management by a dedicated trial manager responsible for day-to-day operations is essential for efficient trial conduct. Indeed, once funding is awarded, the trial manager, rather than the principal investigator and coinvestigators, has been suggested to be the most important research team member to successfully deliver a clinical trial [75]. Furthermore, it is suggested that some trials fail not due to the study design but rather problems with trial management [76,77]. In the context of the ENGAGE feasibility study, no research team member working on the study on a full-time basis had experience of similar research environments and advanced training in research methodology. Those in senior roles with advanced training and expertise in research methodology (eg, the principal investigator and coinvestigator) were key to organizing, planning, and decision-making; however, their time was spread across multiple studies and competing responsibilities. This *light* project management approach has been demonstrated to lead to team members’ expectations being unfulfilled and commitment of the team may decrease [78].

In countries such as the United Kingdom, there has been an increased emphasis on efficient clinical trials to reduce research waste, for example, by the establishment of the UK Trial Managers’ Network for trial managers on academic-led noncommercial trials. To the best of our knowledge, there is no specific trial management professional career structure within academic settings in Sweden, which may account for some of the difficulties recruiting a postdoctoral researcher with the required competencies. An added complexity in the Swedish context is the employment projection law (LAS § 5a 1982:80), which stipulates that personnel can be employed on a fixed-term contract for a maximum of 2 years and must be offered a permanent position thereafter to remain in employment. However, given the time-limited nature of health care research and uncertainty concerning continued funding, this often results in research personnel only being employed for a maximum 2-year fixed-term contract. Subsequently, it is difficult to maintain experienced research staff for the duration of the trial funding periods. However, even in countries such as the United Kingdom, where trial management is more established within academic settings, barriers are experienced, such as a lack of clarity around career structures, progression, and professional status, lack of training opportunities, lack of professional accreditation, and staff retention due to lack of funding and instability of short-term contracts [11,12,79].

A further key challenge related to both the development phase of the internet-administered intervention and digital features to support the execution of study procedures and setup of the feasibility study, occurring concurrently. Complex health care intervention development and refinement involves a number of iterative and interacting stages [19]. The development of interventions using internet-based technologies is complex, and specific challenges have been identified in relation to their development (eg, iterative development life cycles, relationship between academics and developers, and characterization of intervention components and essential features) [80,81]. Furthermore, feasibility studies are complex and can involve a number of iterative phases and the testing of multiple procedural, methodological, and clinical uncertainties [17]. In the present context, a number of challenges were related to the interrelatedness of the intervention and subsequent feasibility study, for example, further development, refinement, and adaptation of the intervention resulted in multiple changes to associated documentation for the feasibility study. The development of high-quality study documentation is a difficult and lengthy process [9], and the focus on intervention development meant allocating resources away from the preparation of study documentation. As such, working on intervention development concurrent with the feasibility study setup may have added to the complexity of already complex research processes.

In addition, while a significant phased approach to intervention development had already taken place [41,42,49,54], a structured approach was not adopted regarding the technical development of the intervention. Given the complexity of the development of internet-administered interventions [80,81], it may have been beneficial to adopt a specific development model designed to inform the development of health care interventions using internet-based technologies, such as the Behavioral Intervention Technology model [82]. Adopting a more structured approach to technical development may have facilitated the communication of technical requirements to the Portal team. Indeed, challenges relating to communication between the research and Portal team may have been experienced, given that requirements were requested from those unfamiliar with software programming, and software developers are unfamiliar with research methodologies and LICBT interventions [23]. The Portal team adopts agile software development, therefore working with software requirements in short iterations [83] with a need for frequent, quick, and short-term decisions [84]. However, this approach can cause challenges when there is a need for collaborative decision-making with multiple stakeholders with varying backgrounds, expertise, and goals [66]. Barriers to efficient communication and development of shared knowledge may be related to a lack of understanding of each other’s field (eg, different educational backgrounds, technical knowledge, and experience), difficulties with clients describing functional requirements, and difficulties with software developers communicating time expectations with clients in a direct manner [68]. A lack of shared understanding between software development teams and end-clients can result in unrealistic planning and frequent changes in planning [66]. In addition, transforming study procedures normally conducted in-person to digital form is a complex procedure requiring significant planning, time, and expertise [25,36]. However, involving a member of the Portal team in the weekly research team meetings significantly improved the development of shared knowledge and understanding and helped to overcome some of the aforementioned challenges. Indeed, more active engagement between software developers and the end client has been posited as a helpful strategy to facilitate communication [68].

Another noteworthy challenge is related to the constraints of the Portal. Health care interventions delivered by digital technologies have evolved rapidly over the past two decades [85]. However, this also means that technology becomes quickly outdated [86], given the technological advancements and changing end-user expectations. However, rapid technical evolution is not easily compatible with traditional research approaches such as randomized controlled trials (RCTs) [85], and translating traditional clinical trial procedures to digital form may require the complete re-engineering of trial design and processes [36]. The Portal was developed to support traditional health care research, for example, RCTs and observational studies, and multiple RCTs of internet-administered cognitive behavioral therapy interventions have been conducted on the Portal [87-90]. The Portal was originally designed to deliver two internet-administered cognitive behavioral therapy interventions [87,88]; however, over time, the Portal has been developed to support the needs of different studies and has experienced a continuous flow of technical requirements from researchers since its conception in 2010 [22,23]. However, the time taken to design and conduct traditional health care research is not in line with fast-paced technical advances [91]. Therefore, when using digital technologies for intervention delivery and the execution of study procedures, careful planning is required as technology may change [25,32]. Multiple requests for new digital features and changes to existing features were required for both the intervention and the execution of study procedures; however, challenges were experienced relating to the legacy software architecture of the Portal. For example, new feature requests or changes to existing features can result in unanticipated negative impacts on existing features used by ongoing studies. This highlights a key challenge for research environments using internet-based technologies, especially in a context where new digital features and complex configurations are added over time [23]. Appropriate technical infrastructure (eg, having the appropriate hardware, software, and technical expertise) has been highlighted as a significant challenge in conducting successful digital health care research [38].

### Limitations

Meeting minutes were written independently to the objective of the analysis, and therefore may not provide sufficient detail to reveal all the challenges experienced by the research team. As is common in document analysis, documents are often uneven in length, providing more detailed information about some topics than others [60]. As such, there is a potential that some challenges experienced were missed in the analysis as less focus was placed on them when writing meeting minutes; however, we attempted to overcome this limitation by member-checking through two group discussions. An additional limitation relates to selectivity bias [60], and it is difficult to separate the analysis from the context of the research team, for example, biases and opinions held by research team members. Attempts to limit selectivity bias were made by the analysis being primarily conducted by a new member of the research team who had not been present in any of the meetings wherein minutes were analyzed.

### Conclusions

The development and feasibility testing of health care interventions using digital technologies is time- and resource-intensive. Recommendations for improving efficiency include (1) the development of networks to share good practice and training opportunities for trial staff, especially in the area of complex digital health care interventions; (2) the employment of advanced research methodology–educated, senior dedicated trial personnel who can be responsible for the day-to-day operations; (3) the completion of the intervention development phase (including technical requirements) before the feasibility study setup; and (4) the integration of members of the software development team into the research team to improve communication and develop shared knowledge and understanding. We hope that our experiences may be useful for others who are planning to conduct future research within the development and feasibility phases of the MRC complex intervention framework, especially for internet-administered interventions and research using digital features to support the execution of study procedures. Publishing challenges experienced during intervention development [19] and trial setup and conduct [11] may help to reduce future research waste, improve the quality of digital health care research, and add to the emerging literature concerning challenges experienced by integrating digital technologies into health care research [25].

Despite experiencing a number of challenges, the ENGAGE feasibility study commenced recruitment on July 3, 2020, and recruitment targets were successfully met by October 14, 2020, well within the projected 6-month recruitment period [21]. Furthermore, preliminary posttreatment follow-up data indicated that retention targets were successfully met. While delays to study start were experienced, taking a careful and detail-orientated approach to intervention development and feasibility study setup may have helped facilitate meeting recruitment and retention targets and will hopefully enhance efficiencies across subsequent phases of our planned research, for example, a definitive (evaluation) trial.

## Abbreviations

LICBT: low-intensity cognitive behavioral therapy
MRC: Medical Research Council
PRP: parent research partner
RCT: randomized controlled trial
